# Treatment of Goitre

**Published:** 1883-09

**Authors:** 


### TREATMENT OF GOITRE.

EVERY improvement in surgery which successfully replaces a large and risk-attended operation by one of a simple and non-dangerous character, must necessarily be regarded in the light of an advance; and in this way a modification of the plan pursued in radical treatment of bronchocele, and which was advocated on Friday last at the meeting of the Clinical Society of London, is freely deserving of general recognition. The operation as described by Mr. Sydney Jones, F. R. C. S., consists in excision of the isthmus of the thyroid instead of extirpation of one or both lobes of the organ; and in a case in which he recently adopted it the proceeding was the most successful possible. A curious consequence of the operation, and on which Mr. Jones laid especial stress, is the invarable atrophy of the gland substances which ensues when the isthmus is taken away; and this fact has not been permitted to pass unnoticed by those Continental surgeons who have led the way in devising and adopting this new operation. That it is new to at any rate a majority of English surgeons, it is fair to assume, since the most recent exposition of English surgery, the revised Holmes "System," is silent in regard to the method. Its obvious advantages, however, so little require to be enforced that no one could be found on Friday to dissent from the opinion, that it offers a manifest mode of simple relief in many instances where the vastly dangerous plan of total extirpation would, but for it, be attempted; and in all cases of small fibrous bronchoceles, which by tightening and pressure produce alarming dyspnœa, the simple and easy operation suggested by Mr. Jones, will in all probability be henceforth received as the only legitimate first proceeding, open to the practical surgeon.

### SUPPORTED BY EVIDENCE.

"Gessler? Who was Gessler?" said Mrs. Beckram to her husband.

"He was a tyrant, my dear, and also a life insurance agent."

"What do you mean by such nonsense?"

"There is no nonsense about it, Mrs. Beckram, I assure you. Does not William Tell say to Gessler in the third act: 'Ha, tyrant, hast thou not given me assurance of my life?' Your husband, madam, never makes a statement that he is not prepared to support by documentary evidence."—*Texas Siftings.*

ROASTED coffee is one of the most powerful disinfectants, not only rendering animal and vegetable effluvia harmless, but really destroying them.

